# Relationship between childhood trauma and non-suicidal self-injury in high school students: the mediating role of the stress perception and the moderating role of teacher-student relationship

**DOI:** 10.1186/s40359-024-01883-7

**Published:** 2024-07-08

**Authors:** Yilin Liu, Yuan Fang, Yuanling Chen, Fuyi Qin, Xinrui Li, Ruibin Feng, Xinyu Luo, Jia Wen, Yatang Chen, Zhaowei Teng, Yong Zeng

**Affiliations:** 1 The Second Affiliated Hospital of Kunming Medical University, Kunming, Yunnan China; 2 School of Public Health and Health Management, Qujing Medical College, Qujing, Yunnan China; 3 Central Laboratory, Chongqing University Central Hospital, Chongqing, China

**Keywords:** Non-suicidal self-injury, Childhood trauma, Stress perception, Teacher-student relationship

## Abstract

This study delves into the correlation between childhood trauma and non-suicidal self-injury (NSSI) behaviors among high school students. Additionally, it examines the mediating role of stress perception and the moderating role of the teacher-student relationship in this association. A questionnaire survey was administered to 1,329 high school students in Yunnan Province to assess childhood trauma, NSSI behaviors, and stress perception. Firstly, the survey revealed a 12% prevalence of NSSI, with girls exhibiting a higher occurrence compared to boys (OR = 0.413, 95% CI: 0.280–0.609). Secondly, childhood trauma emerged as a significant predictor of NSSI behavior, irrespective of gender or whether the individual was an only child (*r* = 0.17, *P* < 0.01). Thirdly, stress perception functioned as a mediator in the relationship between childhood trauma and NSSI among high school students (*t* = 4.65, *P* < 0.01). The mediation effect occupies 26.56% of the total effect. Furthermore, the teacher-student relationship moderated the mediating effect of stress perception on the link between childhood trauma and NSSI (*β* = 0.0736, *P* < 0.01). Notably, individuals with strong teacher-student relationships exhibited a significant elevation in stress perception upon exposure to childhood trauma. The findings of this study support a moderated mediation model in the association between childhood trauma and NSSI, suggesting profound implications for the development of targeted interventions and prevention strategies among high school students.

## Introduction

Non-suicidal self-injury (NSSI) refers to intentional self-harming actions performed by individuals without suicidal intentions, motivated by factors that deviate from societal norms. Typical examples of NSSI behaviors include cutting, scratching, self-hitting, and burning [1]. Survey data suggests that 12.2% of adolescents residing in rural China have engaged in NSSI practices [2]. Additionally, a study conducted among urban adolescents revealed a prevalence rate of 23.2% for NSSI, with 2.3% of these cases exhibiting concurrent suicidal tendencies [3]. A recent Chinese clinical survey, utilizing the DSM-5 diagnostic criteria, found that adolescents aged 13–17 exhibited the highest prevalence of NSSI at 15.9% within the national psychiatric consultation cohort [4]. Current research suggests a trend towards younger age groups engaging in NSSI, with high school students identified as a particularly high-risk population and a prime focus for further investigation. While patients with NSSI are clinically considered to lack suicidal intent, there is a strong correlation between NSSI and potential suicidal behavior risks [5]. Individuals who exhibit severe self-injury often concurrently experience significant suicidal ideation. Furthermore, those engaging in NSSI may be at an increased risk of infection due to inadequate regulation of self-harm severity or delayed medical attention, which could potentially lead to fatal outcomes [6]. Consequently, NSSI poses a significant threat to the lives of high school students and has varying degrees of impact on their physical health.

Exploring the impact of childhood experiences on NSSI among high school students is crucial as these experiences play a pivotal role in shaping fundamental human traits. According to Schema Theory, early childhood experiences are instrumental in establishing schemas that facilitate cognitive functions. Conversely, adverse early experiences can give rise to maladaptive schemas, ultimately leading to undesirable behavioral outcomes [7]. Childhood trauma refers to repeated exposure to physical, emotional, or sexual abuse, or neglect, within familial or societal contexts during childhood and adolescence [8]. A history of childhood trauma predisposes individuals to mental health problems in adulthood and is associated with a range of psychopathologies [9–11]. A meta-analysis has identified significant independent effects of childhood trauma on NSSI [12]. Adolescents who have experienced severe or moderate childhood trauma exhibit a higher prevalence of NSSI behaviors, with those who have undergone severe trauma displaying particularly severe manifestations [13]. Imaging studies have demonstrated structural and functional alterations in the anterior insula among individuals with a history of childhood trauma and those engaging in NSSI. Childhood trauma may cause damage to related brain regions, resulting in impaired emotional regulation and subsequent impulsive NSSI behaviors [14]. Previous studies have established a correlation between childhood trauma and NSSI [15]. However, there is a significant gap in research exploring the mediators and underlying mechanisms that underlie this association [16]. To address this, we introduce a potential mediator that may influence this relationship - stress perception.

Stress perception pertains to individuals’ subjective evaluation of whether various stimulus events and negative factors in their lives are perceived as stressful [17]. This perception, which encompasses individuals’ interpretations of objects and relationships, often triggers feelings of tension and a sense of loss of control. Research has demonstrated that cortisol secretion occurs under stressful conditions, serving to maintain physiological balance. Prolonged exposure to high-stress environments can lead to excessive cortisol secretion, which has deleterious effects on both physical and mental health [18]. This suggests that stress perception serves as a negative predictor of mental health outcomes among adolescents [19]. A recent study exploring the association between real-time assessments of stress and NSSI among young individuals found that higher levels of perceived stress increased the likelihood of successfully resisting NSSI impulses on the same day [20]. This finding aligns with the stress sensitivity theory, which suggests that greater exposure to stressful life events may elevate the risk of engaging in NSSI [21]. This study is grounded in the Resilience Compensation Mechanism model, which posits that positive factors can mitigate the deleterious effects of risk factors on individual outcomes. These positive factors exert a direct and autonomous influence on health, counterbalancing the influence of risk factors [22].

The influence of teacher-student relationships on adolescents is paramount in contemporary society. Drawing from interpersonal suicide theory, feelings of burden and thwarted belonging in key interpersonal interactions may exacerbate suicidal impulses, potentially culminating in self-harm and suicidal behaviors. Significantly, internalized shame or frustration within these relationships can trigger subsequent suicidal ideation and planning [23]. The interpersonal school environment plays a crucial role in the intellectual and psychological development of adolescents [24]. Teachers’ support for students’ psychological needs is instrumental in fostering a positive school environment and climate [25]. Teacher practices that cultivate a sense of support among students, such as affording personal choice and initiative, providing diverse opportunities for success, and establishing close and respectful relationships, can positively impact students’ engagement with NSSI. Conversely, teachers’ negative attitudes and behaviors towards students can influence the occurrence of suicide and NSSI [26].

In summary, to further elucidate the formation and developmental mechanisms underlying NSSI among high school students, a multifactorial integration perspective is required to concurrently investigate the mediating and moderating mechanisms of childhood trauma, stress perception, and teacher-student relationships. The following hypotheses are proposed: H1: Stress perception mediates the relationship between childhood trauma and NSSI in high school students; H2: Teacher-student relationship moderates the mediating effect of childhood trauma on NSSI through stress perception.

## Materials and methods

### Participants

The study randomly selected two high schools in Yunnan Province, China, and conducted standardized tests. These tests were administered and completed by uniformly trained postgraduate students in psychiatry and psychology, with consent obtained from the schools, parents, and subjects (Ethics Clearance Approval No. 2022kmykdx6f47). The participants were informed beforehand of the anonymous and confidential nature of the data collection. All first-year high school students from two randomly selected schools in Yunnan Province, China, were included in the study. Students from higher grades were excluded due to participation in the High School Achievement Test and National College Entrance Examination. After excluding incomplete, careless, and gender-missing questionnaires, a total of 1,329 valid questionnaires were obtained, yielding a validity rate of 94.9%. Among the participants, 513 (38.6%) were male and 816 (61.4%) were female. The participants’ ages ranged from 16 to 20 years, with a mean age of 16.5 years (SD = 0.57).

### Adolescent self-injury questionnaire

The Adolescent Self-Injury Questionnaire, developed by Yu Feng, was utilized as a self-assessment tool to measure NSSI among adolescents. This questionnaire encompassed 18 items and employed a scoring system based on the frequency of self-injurious behavior multiplied by the severity of injury. Self-injury frequency was stratified into 4 levels, while the assessment of bodily injury severity encompassed 5 levels. Adolescents with a total NSSI score ≥ 1 were considered to exhibit NSSI [27]. The Clonbach’s alpha coefficient for this scale in the present study was 0.82.

#### Childhood abuse questionnaire

The Childhood Abuse Questionnaire, originally developed by Bernstein and subsequently revised by Xingfu Zhao et al., comprises 28 items across five subscales: emotional abuse, physical abuse, sexual abuse, emotional neglect, and physical neglect. Each item is rated on a 5-point scale, with three additional items for validity assessment. Higher scores indicate greater abuse experienced during childhood [28]. The internal consistency coefficient of this questionnaire in our study was 0.83. The Stress Perception Scale (SPS), The Chinese version of the PSS-10 scale was employed, consisting of 10 items across two dimensions: crisis perception and coping control ability. Items were rated on a 5-point Likert scale, with four reverse scoring questions. Higher scores, after positive processing of reverse items, indicated a greater perception of stress. The Crisis Perception factor encompassed six negatively described items, while the Coping Control Perception factor included four positively described items [29].

#### Teacher-student relationship questionnaire

The Teacher-Student Relationship Questionnaire, developed by Xinyu Chu from East China Normal University, was employed to assess the teacher-student relationship. This questionnaire comprises 18 questions distributed across three dimensions: the status of the teacher-student relationship, the ease of access in the teacher-student relationship, and the power imbalance in the teacher-student relationship, with six questions per dimension [30]. Each item is rated on a five-point scale, and the total score is calculated by summing the individual item scores. A higher score indicates a more positive teacher-student relationship.

#### Control variables

Previous studies have demonstrated that gender and being an only child significantly impact NSSI [31]. To eliminate the potential confounding effects of these variables on the relationship between the dependent variable and the target variables, they were incorporated as control variables in this study, thereby enhancing the precision of research hypotheses testing.

### Research procedures and data processing

Data were collated and analyzed using SPSS 26.0 and the PROCESS macro program developed by Hayes (2013). To evaluate the mediating process of stress perceptions and the moderating effect of the teacher-student relationship, Model 59 was employed.

## Results

### Common method bias test

A common method bias test utilizing Harman’s one-factor approach revealed the presence of 10 factors with eigenvalues greater than 1 without rotation. Notably, the variance explained by the first factor amounted to 20.71% (<40%) [32]. These findings indicate the absence of significant common method bias in the present study.

### Descriptive statistics and correlation analysis

As presented in Table 1, the teacher-student relationship exhibited a significant negative correlation with stress perception (*r* = -0.398, *P* < 0.01), childhood trauma (*r* = -0.386, *P* < 0.01), and NSSI (*r* = -0.147, *P* < 0.01). Conversely, stress perception demonstrated a significant positive correlation between childhood trauma (*r* = 0.282, *P* < 0.01) and NSSI (*r* = 0.211, *P* < 0.01). Furthermore, childhood trauma was significantly positively associated with NSSI (*r* = 0.17, *P* < 0.01).

**Table 1:** Descriptive statistics and correlations for primary study variables

	M	SD	Teacher-student relationship	Stress perception	Childhood trauma	Non-suicidal self-injury
Teacher-student relationship	2.6332	0.81875	1			
Stress perception	1.857	0.50654	− 0.398**	1		
Childhood trauma	0.5788	0.26411	− 0.386**	0.282**	1	
Non-suicidal self-injury	0.0402	0.18303	− 0.147**	0.211**	0.170**	1

**p < 0.01

### Mediating effect test

A test examining the mediating role of stress perception in the relationship between childhood trauma and NSSI, with gender and being an only child as control variables, revealed the following results (Tables 2 and 3): Childhood trauma significantly positively predicted NSSI (*t* = 6.5574, *P* < 0.01). When stress perception was included as a mediating variable, the positive predictive effect of childhood trauma on NSSI remained statistically significant (*t* = 4.6502, *P* < 0.01). Childhood trauma significantly positively predicted stress perception (*t* = 11.3777, *P* < 0.01), and stress perception also significantly positively predicted NSSI (*t* = 5.634, *P* < 0.01). The 95% bootstrap confidence intervals for both the direct effect of childhood trauma on NSSI and the mediating role of stress perception did not cross zero (Table 3), indicating that childhood trauma is both a direct predictor of NSSI and an indirect predictor via stress perception. The direct effect (0.1297) and indirect effect (0.0469) contributed 73.44% and 26.56% to the total effect (0.1766), respectively, thereby fully supporting H1.

**Table 2:** Mediating model test of stress perception

Regression equation(*N* = 1329)		Overall fit index			Significance of regression coefficient		
Outcome variable	Predictor variable	R	R-sq	F(df)	B	se	t
Non-suicidal self-injury		0.2093	0.0438	20.2382**			
	Gender				0.2348	0.0555	4.2295**
	Only child or not				-0.1351	0.0665	-2.0311*
	Childhood trauma				0.1766	0.0269	6.5574**
Stress perception		0.384	0.1211	60.8478**			
	Gender				0.4209	0.0532	7.9179**
	Only child or not				− 0.0338	0.0637	− 0.5313
	Childhood trauma				0.2935	0.0258	11.3777**
Non-suicidal self-injury		0.2573	0.0662	23.4664**			
	Gender				0.1675	0.0562	2.983**
	Only child or not				-0.1297	0.0658	-1.9721*
	Stress perception				0.1598	0.0284	5.634**
	Childhood trauma				0.1297	0.0279	4.6502**

The standard score was used for each variable in the model. The lower limit of CI and the upper limit of CI refers to the lower and upper limits of the 95% confidence interval (Confidence Interval), respectively. *P < 0.05, **P < 0.01

**Table 3:** Decomposition tables for total, direct and mediated effects

	Effect	BootSE	BootLLCI	BootULCI	Relative effect size
Total effect	0.1766	0.0391	0.1081	0.2621	
Direct effect	0.1297	0.0347	0.0693	0.2039	73.44%
Mediating effects of stress perception	0.0469	0.0118	0.026	0.0726	26.56%

The standard score was used for each variable in the model. The lower limit of CI and the upper limit of CI refers to the lower and upper limits of the 95% confidence interval (Confidence Interval), respectively

### Moderating effect test

**Fig. 1:**
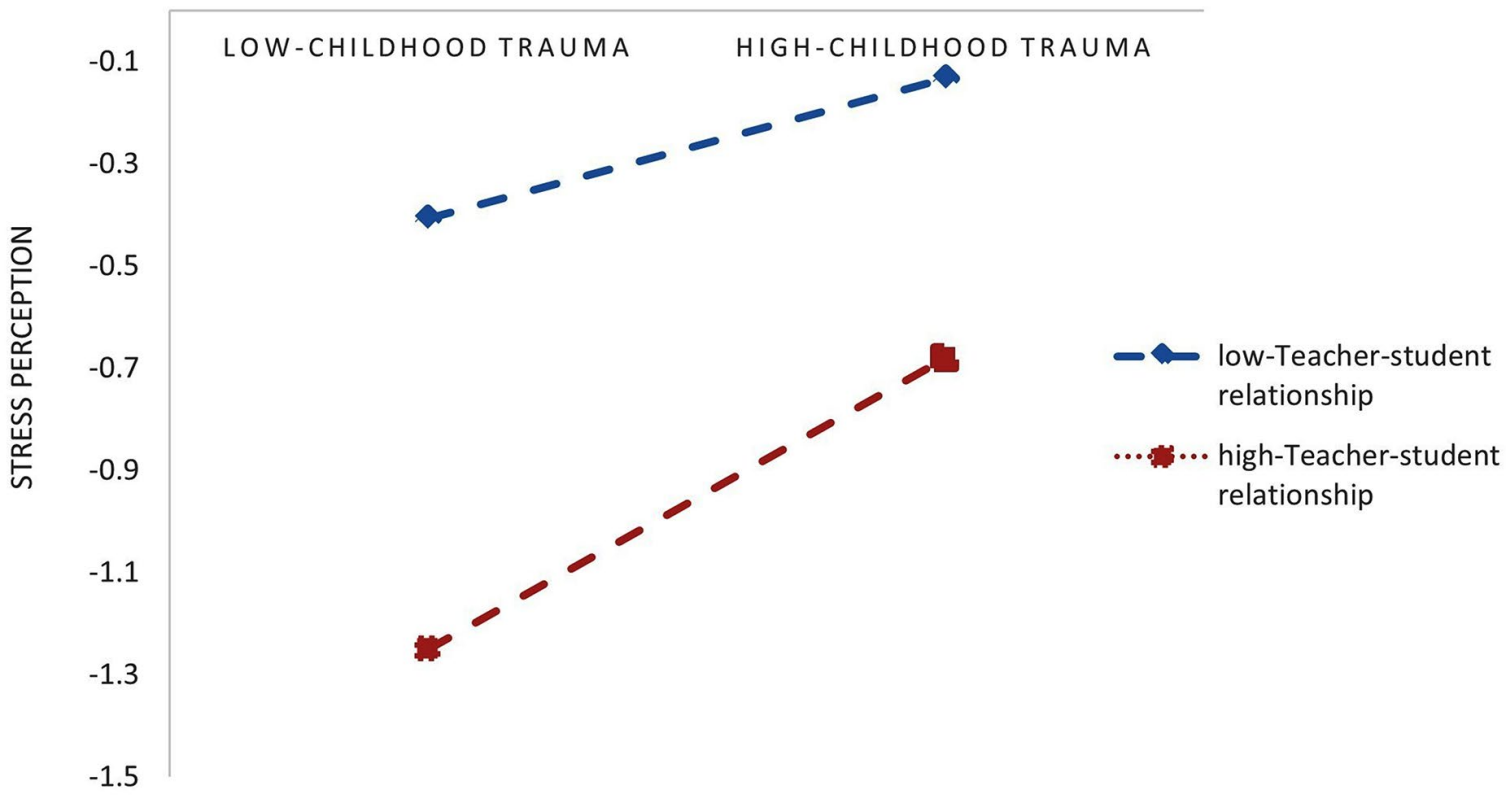
The Moderating Role of Teacher-Student Relationships in the Association Between Childhood Trauma and Perceptions of Stress

The moderating influence of the teacher-student relationship on the association between childhood trauma and stress perception was examined, with gender and being an only child as control variables. Table 4 reveals that the interaction term between childhood trauma and the teacher-student relationship significantly and positively predicted stress perception (*β* = 0.0736, *P* < 0.01), indicating that the predictive effect of childhood trauma on stress perception is moderated by the teacher-student relationship. To further explore the moderating effect of the teacher-student relationship on the association between childhood trauma and stress perception, a simple slope analysis was conducted (Fig. 1). Subgroup regression analysis was conducted, dividing individuals into high and low childhood trauma subgroups based on scores exceeding and falling below the mean plus/minus one standard deviation, respectively. As illustrated in Fig. 1, the results demonstrated that the positive predictive effect of childhood trauma on stress perceptions gradually intensified as the quality of the teacher-student relationship improved. Specifically, the association strengthened from *β* = 0.073 (*P* = 0.0001) to *β* = 0.211 (*P* < 0.001) as the level of the teacher-student relationship increased. Notably, individuals with poorer teacher-student relationships exhibited a significantly higher likelihood of experiencing stress compared to those with stronger relationships. The values about the direct effect of childhood trauma on NSSI and the mediating role of stress perception are presented in Table 5. Furthermore, the mediating effect of stress perception in the association between childhood trauma and NSSI tended to increase across the three levels of the teacher-student relationship. Specifically, as the quality of the teacher-student relationship escalated, childhood trauma was more prone to elicit NSSI behaviors among high school students by heightening their stress perceptions. However, a high level of the teacher-student relationship generally favored lower stress perception levels. These results support H2.

**Fig. 2:**
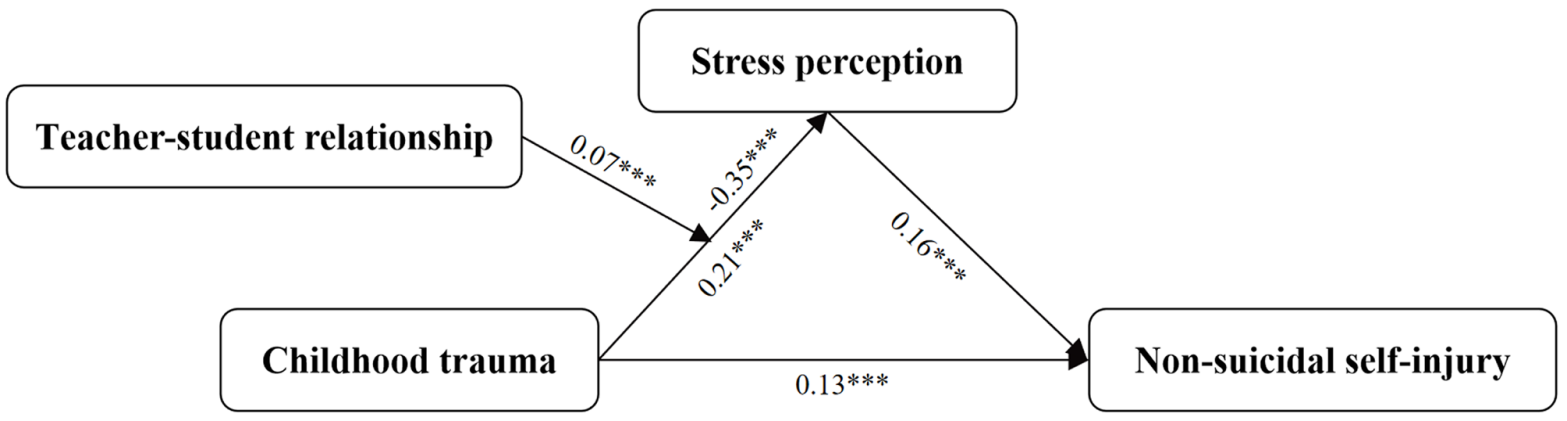
Moderated mediation model regarding perceptions of childhood trauma and NSSI, mediated by stress perception and moderated by teacher-student relationship

In summary, stress perception serves as a mediator in the association between childhood trauma and NSSI. The initial component of this mediating effect is moderated by the teacher-student relationship (Fig. 2). Notably, as the teacher-student relationship strengthens, the mediating role of stress perception does not continuously diminish.

**Table 4:** Moderated mediation model tests

Regression equation(*N* = 1329)		Overall fit index			Significance of regression coefficient		
Outcome variable	Predictor variable	R	R-sq	F(df)	B	se	t
Stress perception		0.4832	0.2335	80.5869**			
	Gender				0.4523	0.0497	9.0939**
	Only child or not				-0.0461	0.0595	-0.7747
	Childhood trauma				0.2111	0.0293	7.203**
	Teacher-Student Relationship				-0.3477	0.0261	-13.3137**
	Childhood trauma						
	×				0.0736	0.019	3.8774**
	Teacher-Student Relationship						
Non-suicidal self-injury		0.2573	0.0662	23.4664**			
	Gender				0.1675	0.0562	2.983**
	Only child or not				-0.1297	0.0658	-1.9721*
	Childhood trauma				0.1297	0.0279	4.6502**
	Stress perception				0.1598	0.0284	5.634**

The standard score was used for each variable in the model. The lower limit of CI and the upper limit of CI refers to the lower and upper limits of the 95% confidence interval (Confidence Interval), respectively. *P < 0.05, **P < 0.01

**Table 5:** Comparison of mediation effects across different levels of the teacher-student relation-ship

	Index	Effect	BootSE	BootLLCI	BootULCI
Moderated mediation effects	eff1(M-1SD)	0.022	0.0071	0.0102	0.0378
	eff2(M)	0.0337	0.0096	0.0176	0.0548
	eff3(M + 1SD)	0.0455	0.0132	0.0235	0.0744
Comparison of Moderated Mediation Effects	eff2-eff1	0.0118	0.0045	0.0039	0.0214
	eff3-eff1	0.0235	0.0089	0.00079	0.0429
	eff3-eff2	0.0118	0.0045	0.0039	0.0214

The standard score was used for each variable in the model. The lower limit of CI and the upper limit of CI refers to the lower and upper limits of the 95% confidence interval (Confidence Interval), respectively

## Discussion

This study established a moderated mediation model within the individual-environment interaction framework, utilizing stress perception as the mediating variable and teacher-student relationship as the moderating variable. This approach elucidated the mechanisms by which childhood trauma influences high school students’ NSSI, mediated by stress perception, and explored the moderating role of teacher-student relationships. The findings hold significant theoretical and practical implications, enhancing our comprehension of the complex interplay between NSSI, individual psychological factors, and adverse relational events. Additionally, these results offer valuable insights for guiding high school students with childhood trauma in managing stressful scenarios and reducing the risk of NSSI. The present study revealed an overall prevalence of 12% for NSSI among the two high schools investigated. We postulate that this finding might be attributed to adolescents’ limited adaptive capacity for internal emotion regulation during this developmental phase [33]. Specifically, adolescents often lack efficacious coping strategies to manage negative emotions, thus predisposing them to resort to NSSI as a means of emotional alleviation. Nevertheless, this behavioral pattern can perpetuate a vicious cycle of escalating psychosocial distress and self-criticism, ultimately impairing social relationships, and amplifying negative emotional states [33]. The prevalence of NSSI was lower in boys than in girls in this study (OR = 0.413, 95% CI [0.280–0.609]), which may be related to the fact that males are more likely to adopt problem-solving strategies when confronted with stressors and negative emotions, whereas females are more likely to focus on emotionally orientated coping strategies [34]. Stress perception, reflecting an individual’s subjective assessment of stress levels, serves as a trigger for anxiety onset [35]. Research indicates a strong link between anxiety and the occurrence of NSSI. Exploring the mediating role of stress perception in NSSI offers insights into how childhood trauma influences its occurrence, potentially informing novel strategies for NSSI prevention.

The results of this study indicate that childhood trauma significantly and positively predicts NSSI in high school students, which is consistent with previous research [33]. Evidence from epidemiology and neurobiology suggests that childhood trauma is strongly associated with enduring brain dysfunction [36]. Drawing from the Experiential Avoidance Model theory, childhood trauma may influence cognitive and behavioral patterns, predisposing individuals to develop a negative self-image and sense of self-worth. Consequently, this heightens the likelihood of engaging in self-injurious behaviors [37]. Childhood trauma can contribute to impaired emotional regulation in adulthood, rendering individuals more vulnerable to daily life stressors and experiencing more intense psychotic reactions [37]. Heightened stress perception increases sensitivity to subsequent adversity, predisposing them to negative moods like anxiety and depression. In the absence of healthy coping mechanisms, individuals may resort to self-injurious behavior as a maladaptive strategy to cope with life’s stresses and negative emotions [38]. In the mediation analysis of this study, childhood trauma was found to influence the occurrence of non-suicidal self-injurious behavior via stress perception, with all path coefficients indicating a positive relationship. These findings suggest a potential mechanism whereby childhood trauma enhances high school students’ sensitivity to stress, subsequently amplifying the impact of pre-existing stressful events and ultimately contributing to the emergence of NSSI. Childhood trauma, an immutable fact, may be evaded or concealed by individuals due to diverse reasons, impeding accurate risk assessment for NSSI in students. Therefore, when assessing the risk of NSSI in a student, it is crucial to consider the potential impact of undisclosed childhood trauma. The current study demonstrates that childhood trauma impacts the occurrence of non-suicidal self-injurious behaviors via stress perception. Consequently, in evaluating the risk of non-suicidal self-injury, it is imperative to consider both the effects of childhood trauma and the level of individual stress perception. The identification of childhood trauma and stress perception as potential risk factors for NSSI has been well-documented in prior studies [12, 21], However, further exploration of these associations and the potential mediators or mitigating factors involved remains crucial.

The present study revealed that the teacher-student relationship moderated the mediating effect of “childhood trauma - stress perception.” This indicates that favorable teacher-student relationships may attenuate the impact of childhood trauma on stress perception, ultimately reducing the occurrence of non-suicidal self-injury. Adolescents exposed to childhood trauma at home may seek support from non-familial relationships, and as high school students spend considerable time at school, it serves as a venue for student-teacher bonding. Positive student-teacher relationships have been associated with improved mental health and social adjustment [39]. Teachers play a pivotal role in supporting students’ fundamental psychological needs by affording them autonomy in making choices during the learning process, facilitating opportunities for self-enhancement, and fostering respectful and intimate relationships among students, school staff, and peers [40]. Research has consistently shown that students who receive teacher support exhibit reduced tendencies toward NSSI [39]. Consequently, to mitigate the risk of NSSI among high school students, a priority should be placed on enhancing the teacher-student relationship.

Our study holds significant theoretical and practical implications for intervening in NSSI among high school students. It theoretically enhances the understanding of childhood trauma’s influence on NSSI, exploring potential mechanisms involving stress perception and teacher-student relationships. Additionally, it offers a theoretical framework and novel perspective for interventions, enabling teachers to effectively leverage positive emotions in their educational practices to foster students’ psychological well-being. Childhood trauma indirectly impacts high school students’ NSSI through the mediating factor of stress perception, which can be modulated by the teacher-student relationship. The findings of this study further indicate that schools and teachers should prioritize interventions for high school students exhibiting NSSI behaviors by integrating individual stress perception and teacher-student relationships. Attention should be paid to students’ childhood traumatic experiences, fostering adaptive stress perception and emotion regulation, and strengthening teacher-student relationships to mitigate the risk of NSSI. The results of this study shed light on the complex interactions between individual psychological factors, adverse relationship events, and NSSI. Prior research often focused on isolated factors [12–16, 20], whereas this study provides a more holistic view by integrating both individual and environmental variables. However, despite the progress made in this study, there are still some limitations. Firstly, the exclusive use of self-report methodology may have introduced biases in the results. Future research could enhance the reliability and validity of findings by employing multiple methods, including self-report, parent report, and teacher assessment, to measure the key variables. Secondly, a cross-sectional study design precludes the establishment of causal inferences. Therefore, the moderated mediation model proposed in this study could be further validated in future longitudinal studies. In the present study, stress perception was measured using the Stress Perception Scale, primarily conceptualizing it as a trait. However, attention to the dynamic processes of stress perception was lacking. Future research could experimentally manipulate stress perception in diverse ways to elicit distinct stress perception processes among individuals. This approach would allow for a more nuanced examination of the moderating role of stress perception in the relationships between childhood trauma, teacher-student relationships, and NSSI. Lastly, the sample of this study comprised high school students from two specific high schools, potentially limiting the generalizability of the findings. Future researchers could enhance the external validity of the study by selecting high school students from diverse geographical areas to further test the primary findings of this study.

## Data Availability

No datasets were generated or analysed during the current study.

## References

[1] Brown RC, Plener PL (2017). Non-suicidal Self-Injury in Adolescence. Curr Psychiatry Rep Mar.

[2] Tang J, Li G, Chen B (2018). Prevalence of and risk factors for non-suicidal self-injury in rural China: results from a nationwide survey in China. J Affect Disorders Jan 15.

[3] Liang S, Yan J, Zhang T (2014). Differences between non-suicidal self injury and suicide attempt in Chinese adolescents. Asian J Psychiatry Apr.

[4] Guo C, Tomson G, Keller C, Söderqvist F (2018). Prevalence and correlates of positive mental health in Chinese adolescents. BMC Public Health Feb.

[5] Hamza CA, Stewart SL, Willoughby T (2012). Examining the link between nonsuicidal self-injury and suicidal behavior: a review of the literature and an integrated model. Clin Psychol Rev Aug.

[6] Lewis SP, Heath NL, Michal NJ, Duggan JM (2012). Non-suicidal self-injury, youth, and the internet: what mental health professionals need to know. Child and adolescent psychiatry and mental health. Mar 30.

[7] Temple SD (2003). Schema Therapy: a practitioner’s guide. Am J Psychiatry.

[8] Zhang S, Lin X, Liu J (2020). Prevalence of childhood trauma measured by the short form of the Childhood Trauma Questionnaire in people with substance use disorder: a meta-analysis. Psychiatry Res Dec.

[9] McGrath JJ, McLaughlin KA, Saha S (2017). The association between childhood adversities and subsequent first onset of psychotic experiences: a cross-national analysis of 23 998 respondents from 17 countries. Psychol Med May.

[10] Huh HJ, Kim KH, Lee HK, Chae JH (2017). The relationship between childhood trauma and the severity of adulthood depression and anxiety symptoms in a clinical sample: the mediating role of cognitive emotion regulation strategies. J Affect Disorders Apr 15.

[11] Destrée L, Brierley ME, Albertella L, Jobson L, Fontenelle LF (2021). The effect of childhood trauma on the severity of obsessive-compulsive symptoms: a systematic review. J Psychiatr Res Oct.

[12] Liu RT, Scopelliti KM, Pittman SK, Zamora AS (2018). Childhood maltreatment and non-suicidal self-injury: a systematic review and meta-analysis. Lancet Psychiatry Jan.

[13] Wang L, Zou HO, Liu J, Hong JF. Prevalence of adverse childhood experiences and their associations with Non-suicidal Self-Injury among Chinese adolescents with Depression. Child psychiatry and human development. Feb. 2023;22. 10.1007/s10578-023-01508-x. 10.1007/s10578-023-01508-x 36811752

[14] Deng W, Long J, Liu L (2023). Research Progress on Magnetic Resonance Imaging in Childhood Trauma and Non-suicidal Self-Injury. J Int Psychiatry.

[15] Zhang JJ, Liu YD, Zhang H (2022). Correlates of non-suicidal Self-Injury in Adolescent Psychiatric patients in China. Front Psychiatry.

[16] Serafini G, Canepa G, Adavastro G (2017). The relationship between Childhood Maltreatment and Non-suicidal Self-Injury: a systematic review. Front Psychiatry.

[17] Li Y, Li X, Li J (2021). Application of the Chinese version of the stress perception scale in representative community adult population. Chin Mental Health J.

[18] Nishimi K, Koenen KC, Coull BA, Segerstrom SC, Austin SB, Kubzansky LD (2022). Psychological resilience and diurnal salivary cortisol in young adulthood. Psychoneuroendocrinology Jun.

[19] Xu C, Peng L, Fu W, Liu X, Zhang L, Li M (2022). Path analysis of perceived stress affecting mental health problems in adolescents. Chongqing Med.

[20] Turner BJ, Baglole JS, Chapman AL, Gratz KL. Oct. Experiencing and resisting nonsuicidal self-injury thoughts and urges in Everyday Life. Suicide & life-threatening behavior. 2019;49(5):1332–46. 10.1111/sltb.12510. 10.1111/sltb.12510 30152181

[21] March-Llanes J, Marqués-Feixa L, Mezquita L, Fañanás L, Moya-Higueras J (2017). Stressful life events during adolescence and risk for externalizing and internalizing psychopathology: a meta-analysis. Eur Child Adolesc Psychiatry Dec.

[22] Zimmerman MA (2013). Resiliency theory: a strengths-based approach to research and practice for adolescent health. Health Educ Behavior: Official Publication Soc Public Health Educ Aug.

[23] O’Connor RC, Kirtley OJ. The integrated motivational-volitional model of suicidal behaviour. Philosophical transactions of the Royal Society of London Series B, Biological sciences. Sep. 2018;5(1754). 10.1098/rstb.2017.0268. 10.1098/rstb.2017.0268 PMC6053985 30012735

[24] Eccles JS, Roeser RW (2011). Schools as Developmental contexts during Adolescence. J Res Adolescence.

[25] Roth G, Kanat-Maymon Y, Bibi U (2011). Prevention of school bullying: the important role of autonomy-supportive teaching and internalization of pro-social values. Br J Educational Psychol Dec.

[26] Nguyen TH, Nguyen HMT, Ha TT, Nguyen NN (2022). The role of teacher and peer support against bullying among secondary School students in Vietnam. J Genetic Psychol Sep-Oct.

[27] Yu F. Adolescent self-injurious behaviour in relation to individual emotional and family environmental factors. Central China Normal University; 2008.

[28] Xingfu Z. Reliability and validity of the childhood abuse questionnaire and the psychosocial and molecular biology of male somatic abusers of domestic violence. Central South University; 2005.

[29] Liu W, Yi J, Zhong T, Zhu X (2015). Measurement invariance of the perceived stress scale in College men and women. Chin J Clin Psychol.

[30] Chu X. Experimental Research in Influence of Different Climates on Junior High School Students’ Learning Interests of Physical Education and Relationship between Students and Teachers. East China Normal University; 2006.

[31] Wang S, Xu H, Zhang S, Wan Y, Tao F (2020). Mediating effects of self-esteem in the relationship between childhood maltreatment and non-suicidal self-injury among adolescents: the roles of sex and only-child status. Social Sci Med Feb.

[32] Jia J, Li D, Li X, Zhou Y, Wang Y, Sun W (2017). Psychological security and deviant peer affiliation as mediators between teacher-student relationship and adolescent internet addiction. Comput Hum Behav.

[33] Wang YJ, Li X, Ng CH, Xu DW, Hu S, Yuan TF (2022). Risk factors for non-suicidal self-injury (NSSI) in adolescents: a meta-analysis. EClinicalMedicine Apr.

[34] Meléndez JC, Mayordomo T, Sancho P, Tomás JM (2012). Coping strategies: gender differences and development throughout life span. Span J Psychol.

[35] Liao H, Du J, Xiao R (2024). Relationship among anxiety, perceived stress and forbearance in college students. Chin Mental Health J.

[36] Herzog JI, Schmahl C (2018). Adverse childhood experiences and the consequences on Neurobiological, Psychosocial, and somatic conditions across the Lifespan. Front Psychiatry.

[37] Chapman AL, Gratz KL, Brown MZ (2006). Solving the puzzle of deliberate self-harm: the experiential avoidance model. Behav Res Therapy Mar.

[38] Lardinois M, Lataster T, Mengelers R, Van Os J, Myin-Germeys I (2011). Childhood trauma and increased stress sensitivity in psychosis. Acta Psychiatrica Scand Jan.

[39] Forster M, Gower AL, Borowsky IW, McMorris BJ (2017). Associations between adverse childhood experiences, student-teacher relationships, and non-medical use of prescription medications among adolescents. Addict Behav May.

[40] Maarten V, Bart PNC (2010). The development of the five mini-theories of self-determination theory: an historical overview, emerging trends, and future directions. Adv Motivation Achievement.

